# DNA damage and sequence specificity of DNA binding of the new anti-cancer agent 1,4-bis(2'-chloroethyl)-1,4-diazabicyclo-[2.2.1] heptane dimaleate (Dabis maleate)

**DOI:** 10.1038/bjc.1990.53

**Published:** 1990-02

**Authors:** M. Broggini, J. A. Hartley, W. B. Mattes, M. Ponti, K. W. Kohn, M. D'Incalci

**Affiliations:** Laboratory of Cancer Chemotherapy, Istituto di Ricerche Farmacologiche Mario Negri, Milan, Italy.

## Abstract

The DNA damage and the sequence specificity of guanine-N7 alkylation produced by the novel, positively charged, antineoplastic agent 1,4-bis(2'-chloroethyl)-1,4-diazabicyclo-[2.2.1] heptane dimaleate (Dabis maleate) and its uncharged tertiary amine analogue 1,4-bis(2'-chloroethyl)-1,4-diazacyclohexane (Dabis analogue) were investigated in L1210 cells and isolated DNA. Both compounds are cytotoxic in vitro causing an arrest of L1210 cells in G2/M phase of the cell cycle. In isolated DNA, Dabis maleate alkylates guanine at the N7-position with some differences in specificity compared to other alkylating agents (e.g. nitrogen mustard). Significant differences are also evident between Dabis maleate and Dabis analogue, suggesting that Dabis analogue is not the sole alkylating species of Dabis maleate. Using the alkaline elution technique a moderate number of DNA interstrand cross-links were detected in L1210 cells treated with both compounds, which were completely repaired within 24 h. Dabis maleate and Dabis analogue do not cause DNA single strand breaks or DNA protein cross-links at the doses at which DNA interstrand cross-links were detected.


Br. J. Cancer (1990), 61, 285-289 © Macmillan Press Ltd., 1990

### DNA damage and sequence specificity of DNA binding of the new anti-cancer agent 1,4-*bis*(2′-chloroethyl)-1,4-diazabicyclo-[2.2.1] heptane dimaleate (Dabis maleate)

M. Broggini[1], J.A. Hartley[2,*], W.B. Mattes[2,†], M. Ponti[1], K.W. Kohn[2] & M. D'Incalci[1]

[1]*Laboratory of Cancer Chemotherapy, Istituto di Ricerche Farmacologiche Mario Negri, via Eritrea 62, Milan, Italy; and*
[2]*Laboratory of Molecular Pharmacology, DTP, DCT, National Cancer Institute, NIH, Bethesda, MD, USA.*

**Summary** The DNA damage and the sequence specificity of guanine-N7 alkylation produced by the novel, positively charged, antineoplastic agent 1,4-*bis*(2′-chloroethyl)-1,4-diazabicyclo-[2.2.1] heptane dimaleate (Dabis maleate) and its uncharged tertiary amine analogue 1,4-*bis*(2′-chloroethyl)-1,4-diazacyclohexane (Dabis analogue) were investigated in L1210 cells and isolated DNA. Both compounds are cytotoxic *in vitro* causing an arrest of L1210 cells in G2/M phase of the cell cycle. In isolated DNA, Dabis maleate alkylates guanine at the N7-position with some differences in specificity compared to other alkylating agents (e.g. nitrogen mustard). Significant differences are also evident between Dabis maleate and Dabis analogue, suggesting that Dabis analogue is not the sole alkylating species of Dabis maleate. Using the alkaline elution technique a moderate number of DNA interstrand cross-links were detected in L1210 cells treated with both compounds, which were completely repaired within 24 h. Dabis maleate and Dabis analogue do not cause DNA single strand breaks or DNA protein cross-links at the doses at which DNA interstrand cross-links were detected.

1,4-*bis*(2′-chloroethyl)-1,4-diazabicyclo-[2.2.1] heptane dimaleate (Dabis maleate, NSC 262666, see structure in Figure 7a) is one of a series of quaternary ammonium salts which has been synthesised (Fessler *et al.*, 1969) and tested for anti-tumour activity. Dabis maleate and Dabis perchlorate were found to be active in several tumour systems of mice and rats, including L1210 leukaemia, colon 26, Lewis lung carcinoma and Walker 256 carcinosarcoma (Pettit *et al.*, 1979). The activity of the two drugs was similar, but since Dabis perchlorate is potentially explosive, further studies were conducted with Dabis maleate.

In order to elucidate the mode of action of this drug, which is undergoing phase I clinical trials, we undertook this study in which we characterised the DNA damage in intact cells and the sequence specificity of guanine-N7 alkylation in isolated DNA.

These studies were conducted in parallel with the uncharged tertiary amine 1,4-*bis*(2′-chloroethyl)-1,4-diazacyclohexane (Dabis analogue, see structure in Figure 7c) lacking the bridge between the two nitrogen atoms to determine to what extent the positive charges present in the Dabis maleate molecule could play a role in the mechanism of action of this drug. A comparison of the pattern of N7-guanine alkylation of Dabis maleate and Dabis analogue could give an indication of the mechanism of alkylation of this novel anti-cancer agent.

### Materials and methods

### *Drugs*

Dabis maleate and Dabis analogue were obtained from Dr Winograd of the New Drug Developmental Office (Amsterdam) and from the Drug Developmental Program, Division of Cancer Treatment (NCI, Bethesda, Maryland).

Plasmid DNAs and restriction endonucleases were purchased from Bethesda Research Laboratories; $\gamma^{32}$P-ATP (specific activity 5,000 Ci mmol$^{-1}$) was obtained from Amersham. All the other reagents were of the greatest available purity.

### *Cell cytotoxicity*

Exponentially growing L1210 cells were treated with different concentrations of Dabis maleate and Dabis analogue for 1 h at 37°C. At the end of treatment cells were washed with phosphate-buffered saline (PBS) and resuspended in drug-free medium. After 48 h cells were diluted with fresh medium to maintain the concentration between 0.3 and $1.5 \times 10^6$ cells ml$^{-1}$. In this range the growth of control cells is logarithmic. The effect of drugs on cell growth was evaluated after recovery times of 24, 48 and 72 h using a Coulter Counter model ZB (Kontron).

The effects of drug treatment on cell cycle phase distribution were evaluated after a recovery time of 24, 48 and 72 h using a 30-L cytofluorograph (Ortho Diagnostic System, USA). One ml of L1210 cells was centrifuged for 10 min at 1,200 r.p.m. and directly stained with 1 ml of propidium iodide (PI) solution (Calbiochem Behring, USA) containing 50 μg ml$^{-1}$ of PI in 0.1% sodium citrate and 7.5 μg of RNase (Calbiochem Behring, USA) in water at room temperature for 15 min (Erba *et al.*, 1986).

The fluorescence pulse was detected in a spectral range between 580 and 750 nm; the coefficient of variation of the G1 peak of the L1210 cells was about 4–5%. Each cytofluorimetric assay was performed with $1-2 \times 10^4$ cells.

### *Sequence specificity of guanine-N7 alkylation*

The method has been previously described in detail (Mattes *et al.*, 1986). Briefly, Bam HI digested pBR322 DNA was $^{32}$P-labelled at its 5′ ends with T4 polynucleotide kinase and $\gamma^{32}$P-ATP (Maxam & Gilbert, 1980). A second cut with Sal I was performed to produce a 276 bp fragment labelled at only one end which was isolated from agarose gels. Alkylation was performed in 25 mM triethanolamine, 1 mM EDTA, pH 7.2, at 37°C or room temperature for different times at doses selected to give approximately one alkylation per DNA molecule. After precipitation and washing the DNA was treated for 15 min at 90°C with 1 M piperidine to produce breaks specifically at sites of guanine-N7 alkylation.

DNA fragments were separated on 0.4 mm, 6% polyacrylamide gels containing 7 M urea and a Tris-boric acid-EDTA buffer system. Gels were run at 85 W (approximately 3,500 V) for 3 h. Following autoradiography of the gel, relative band intensities were determined by microdensitometry using a Beckman DU-8 scanning spectrophotometer with gel scanning accessory.

*Present address: Department of Oncology, University College and Middlesex School of Medicine, London, UK.
†Present address: ICI Amencas Inc., Agricultural Products Environmental Health Center, Farmington, CT, USA.
Correspondence: M. Broggini.
Received 24 April 1989; and in revised form 4 October 1989.

*DNA damage in intact cells*

L1210 cells growing in RPMI 1640 were labelled with $^3$H-thymidine (specific activity 20 Ci mmol$^{-1}$; NEN) for 24 h at a concentration of 0.1 $\mu$Ci ml$^{-1}$ in medium containing $10^{-6}$ M unlabelled thymidine. After 16–24 h chasing in medium without $^3$H-thymidine, cells were treated for 1 h with 400 $\mu$M Dabis maleate or 4 $\mu$M Dabis analogue. At the end of treatment or after 6 and 24 h of post incubation in drug-free medium, DNA single strand breaks (DNA-SSB), DNA interstrand cross-links (DNA-ISC) and DNA–protein cross-links were determined by the alkaline elution technique (Kohn *et al.*, 1981).

For the DNA-SSB, cells were washed in ice cold PBS and layered on polycarbonate filters, 0.8 $\mu$m pore size and 25 mm diameter. Cells were then lysed with a solution containing 2% sodium dodecyl sulphate, 0.02 M EDTA, 0.1 M glycine, pH 10.0 (lysis solution), which was allowed to flow through the filter by gravity. After connection of the outlet of the filter holders to the pumping system, 2 ml of proteinase K (0.5 mg ml$^{-1}$ dissolved in lysis solution) were added to a reservoir over the polycarbonate filters and pumped for approximately 1 h at a rate of 0.35 ml min$^{-1}$. DNA was eluted from the filters by pumping 0.02 M EDTA solution adjusted to pH 12.1 with tetrapropylammonium hydroxide containing 0.1% sodium dodecyl sulphate through the filters at approximately 2 ml h$^{-1}$. Fractions were collected 3-hourly, with fractions and filters processed as described (Kohn *et al.*, 1981).

For the DNA–protein cross-links, cells were layered on DM 800 Metricel filters, 2 $\mu$m pore size and 25 mm diameter and then lysed with 5 ml of 2 M NaCl, 0.04 M EDTA, 0.2% *N*-laurylsarcolisine, pH 10.0. The detergent was then washed away with 5 ml of 0.02 M EDTA solution. The elution buffer was the same as that used for DNA single-strand breaks, pH 12.1, except that no sodium dodecyl sulphate was added.

For the DNA-ISC, cells were irradiated with 450 rad at 0°C and then layered on filters and processed as described for the DNA-SSB.

### Results

Figure 1 shows the cell growth inhibition effect on L1210 cells exposed *in vitro* for 1 h at different concentrations of Dabis maleate and Dabis analogue and evaluated after recovery times of 24, 48 and 72 h. Both drugs cause an inhibition of cell growth in a dose-dependent manner with Dabis analogue showing the same effects as Dabis maleate but at 200-fold lower concentrations.

**Figure 1** Cell growth inhibition of different doses of Dabis maleate and Dabis analogue at the concentrations indicated in the figure after 1 h treatment of L1210 cells. –◆– Control; –O– Dabis maleate 200 $\mu$M; –■– Dabis maleate 400 $\mu$M; –□– Dabis maleate 800 $\mu$M; –▲– Dabis analogue 1 $\mu$M; –△– Dabis analogue 2 $\mu$M; –O– Dabis analogue 4 $\mu$M.

The effects of Dabis maleate and Dabis analogue on cell cycle phase distribution are shown in Figure 2. One hour treatment with both compounds induces an accumulation of cells in late S-G2M phases (SL-G2M) that, for the highest dose (Dabis maleate 800 $\mu$M and Dabis analogue 4 $\mu$M), is still evident after 48 h recovery time, but reverting to normal after 72 h. Again, Dabis analogue shows the same pattern of cell cycle arrest at concentrations that are 200 times lower than those required for Dabis maleate.

In order to determine whether or not the alkylation reactions of Dabis maleate and of Dabis analogue are due to the same reactive species, the intensity patterns for reactions with guanines in a defined DNA segment were compared. If the proximal reactive species are the same, then the reaction intensity patterns should be identical. Figure 3a and c shows that there are significant differences between the two com-

**Figure 2** Cell-cycle phase distribution of L1210 cells *in vitro* exposed for 1 h at 37°C, at the concentrations indicated, to Dabis maleate and Dabis analogue and evaluated after 24, 48 and 72 h recovery.

**Figure 3** Densitometric scans of guanine-N7 alkylation produced in the 276 bp Bam HI-Sal I fragment of pBR322 5′ end labelled at Bam HI site. Panel **a**, 1 mM Dabis maleate for 1 h at room temperature. **b**, 100 μM Dabis maleate for 1 h at 37°C. **c**, 1 μM Dabis analogue for 1 h at room temperature. The correspondence between nucleotide positions 458 and 549 is shown along the abscissa. Arrows point to some major differences between the patterns.

pounds at certain sites. The most notable difference is at guanine 524 (for sequence see Figure 4), which, relative to other guanines, reacts much more intensely with Dabis analogue than with Dabis maleate (arrow in Figure 3c). Guanines 536, 537 and 538 react more weakly with Dabis analogue than with Dabis maleate (arrows in Figure 3a). In addition, the major peaks located at the central G at position 472 and at position 486 are relatively weaker for Dabis maleate than for Dabis analogue; in both cases the sequence about the central G is 5′-CGGGC-3′.

The above reactions were carried out at room temperature at doses which gave comparable levels of alkylation. When Dabis maleate was reacted at 37°C, the intensity pattern was shifted part of the way toward that seen with Dabis analogue (Figure 3b). The reason for this will be considered in the Discussion. By contrast the reaction pattern produced by Dabis analogue was essentially identical at room temperature and at 37°C (data not shown). For the purpose of orientation relative to a standard nitrogen mustard, the intensity pattern for reaction with *N,N-bis*(2-chloroethyl)-*N*-methylamine (HN2) in the same DNA region is shown in Figure 5. The pattern is similar (although not identical) to that obtained for Dabis maleate at room temperature (Figure 3a).

Figure 6 shows the formation of DNA-ISC in L1210 cells treated for 1 h with 400 μM Dabis maleate and 4 μM Dabis analogue. Both compounds produced slight, but significant, DNA-ISC at the end of treatment which were still present after 6 h of post incubation in drug-free medium (approximately 30–50 rad equivalents). By 24 h no DNA-ISC were evident, suggesting that the few DNA-ISC formed were already repaired.

Neither compound produced any detectable DNA-SSB or DNA–protein cross-links (data not shown) as assessed by alkaline elution at the doses at which DNA-ISC were detected and at which significant growth inhibition was observed.

**Figure 4** DNA sequence showing the locations of major differences between Dabis maleate and Dabis analogue. The up arrow indicates higher reaction intensity in Dabis analogue than in Dabis maleate; down arrows indicate the reverse.

**Figure 5** Densitometric scan of guanine-N7 alkylation produced by 20 μM HN2 for 1 h at room temperature. DNA fragment analysed is the same as in Figure 3 but is from a separate gel.

**Figure 6** Formation of DNA-ISC in 1210 cells treated with Dabis maleate (400 μM) or Dabis analogue (4 μM) for 1 h and evaluated at different recovery times as indicated. All the samples were irradiated with 450 rad before alkaline elution. Controls are not irradiated cells. For details see Materials and methods. –◇– Control; –●– 450 rad; –□– Dabis maleate 0h; –○– Dabis maleate 6 h; –■– Dabis maleate 24 h; –◆– Dabis analogue 0h; –▲– Dabis analogue 6 h; –△– Dabis analogue 24 h.

### Discussion

We confirm that Dabis maleate is cytotoxic against L1210 leukaemia cells, causing an arrest in late S or G2 phase of the cell cycle (Traganos *et al.*, 1984). The induction of a block in late S or G2 is a common feature of many DNA damaging antineoplastic drugs and would suggest that Dabis maleate and the more toxic Dabis analogue interact with DNA.

Although direct alkylation by Dabis maleate is unexpected, since chemical mechanisms would not predict alkylating reactivity of the quaternary amino groups, we found that Dabis maleate did lead to alkylation of guanine-N7 positions in isolated DNA. The low potency of Dabis maleate compared to other alkylating agents such as nitrogen mustard suggested the possibility that the compound may convert chemically to a tertiary amine form with alkylating activity. A chemically plausible mechanism (Pettit *et al.*, 1979) would be the loss of the bridging $CH_2$ as formaldehyde to yield the tertiary amine Dabis analogue (Figure 7c). If this were the sole alkylating species generated, then the reactivity pattern for Dabis maleate should be identical to that of Dabis analogue. We found, however, that the two compounds exhibit clear differences in the pattern of guanine-N7 alkylation intensities in a DNA sequence. This excludes the possibility that the proximal reactive species produced by Dabis maleate and Dabis analogue are identical.

A simple hypothesis proposed here is that the loss of the bridging $CH_2$ from Dabis maleate occurs in two steps and that an intermediate, possibly an aldehyde, as indicated in Figure 7b, or a hydroxymethyl derivative, persists long enough to contribute significantly to the alkylation reactions. The hypothesised intermediate has both a tertiary and a quaternary 2-chloroethylamino group, and the tertiary group should be able to initiate alkylation reactions.

The aldehyde (or hydroxymethyl) group in Figure 7b would be present in the transition state of the alkylation reaction and therefore could affect the relative reaction rates at different guanines, depending upon the DNA sequence environment. This mechanism provides an explanation for the temperature dependence of the sequence selectivity of Dabis maleate seen in Figure 3. At the higher temperature (37°C), the intermediate species would decompose faster to Dabis analogue (Figure 7c) and therefore would contribute less to the overall reaction. This would be in accord with the finding that, at the higher temperature, there is less difference between the reaction patterns of Dabis maleate and Dabis analogue. The pattern of guanine-N7 alkylation of Dabis maleate is similar to, but not the same as, that of nitrogen mustard. For example, alkylation of three adjacent guanines in a run of five guanines is much greater than for the nitrogen mustard, which produces intense alkylation in only one of the five guanines.

As previously observed for nitrogen mustards (Ewig & Kohn, 1977; Ross *et al.*, 1978) no DNA-SSB were detected in L1210 cells treated with Dabis maleate or Dabis analogue. In contrast to nitrogen mustard, however, no DNA–protein cross-links were detected at doses at which DNA-ISCs were produced. The only lesions detected at doses which produced an inhibition of cell growth were DNA-ISCs suggesting that they were cytotoxic to the cells. The nature of the low level of DNA-ISCs produced remains to be determined, and may differ from that of those produced by other alkylating agents, such as nitrogen mustards, because of the differing distance between the two alkylating moieties within the drug molecules.

c

**Figure 7** Chemical structure of Dabis maleate (**a**) and Dabis analogue (**c**). Compound (**b**) is the possible reactive species of Dabis maleate.

In conclusion, the novel agent Dabis maleate appears to be similar to conventional bifunctional alkylating agents such as nitrogen mustards in that it produces DNA-ISC and a cell cycle arrest in G2M at pharmacologically relevant doses, but differs in that it produces a different pattern of guanine-N7 alkylation in purified DNA and no evidence of DNA–protein cross-links in cells. The tertiary amine Dabis analogue, which was found to be very toxic *in vivo* (Pettit *et al.*, 1979), was active *in vitro* at 200-fold lower concentrations, but the different patterns of alkylation produced by this compound suggest that this is not the sole alkylating species of Dabis maleate.

The generous contribution of the Italian Association for Cancer Research, Milan, Italy, is gratefully acknowledged. Partially supported by CNR (PF Biotecnologie e Biostrumentazione No. 89/0024470) and by Progetto Bilaterale, 1989.

### References

ERBA, E., PEPE, S., UBEZIO, P. & 5 others (1986). Mitozolomide activity on human cancer cells *in vitro*. *Br. J. Cancer*, **54**, 925.

EWIG, R.A.G. & KOHN, K.W. (1977). DNA damage and repair in mouse leukemia L1210 cells treated with nitrogen mustard, 1,3-*bis*(2-chloroethyl)-1-nitrosourea, and other nitrosoureas. *Cancer Res.*, **37**, 2114.

FESSLER, D.C., PETTIT, G.R. & SETTEPANI, J.A. (1969). Antineoplastic agents. XXV. 1,4-Diazabicyclo-[2.2.1]-heptanes. *J. Med. Chem.*, **12**, 542.

KOHN, K.W., EWIG, R.A.G., ERICKSON, L.C. & ZWELLING, L.A. (1981). Measurement of strand breaks and cross-links by alkaline elution. In *DNA Repair. A Laboratory Manual of Research Procedures, Vol.1 pt. B*, Freidberg, E.C. & Hanawalt, P.C. (eds) p. 379. Marcel Dekker: New York.

MATTES, W.B., HARTLEY, J.A. & KOHN, K.W. (1986). DNA sequence selectivity of guanine-N7 alkylation by nitrogen mustards. *Nucleic Acids Res.*, **14,** 2971.

MAXAM, A.M. & GILBERT, W. (1980). Sequencing end-labeled DNA with base-specific chemical cleavage. *Methods Enzymol.*, **65,** 499.

PETTIT, G.R., GIESCHEN, D.P. & PETTIT, W.E. (1979). Antineoplastic agents LXIV: 1,4-*bis*(2′-chloroethyl)-1,4-diazabicyclo-[2.2.1]heptane dihydrogen, dimaleate. *J. Pharm. Sci.*, **68,** 1539.

ROSS, W.E., EWIG, R.A.G. & KOHN, K.W. (1978). Differences between melphalan and nitrogen mustard in the formation and removal of DNA cross-links. *Cancer Res.*, **38,** 1502.

TRAGANOS, F., DARZYNKIEWICZ, Z., BUETI, C. & MELAMED, M.R. (1984). Effects of a prospective antitumour agent, 1,4-*bis*(2′chloroethyl)-1,4-diazabicyclo-[2.2.1] heptane diperchlorate, on cultured mammalian cells. *Cancer Invest.*, **2,** 1.


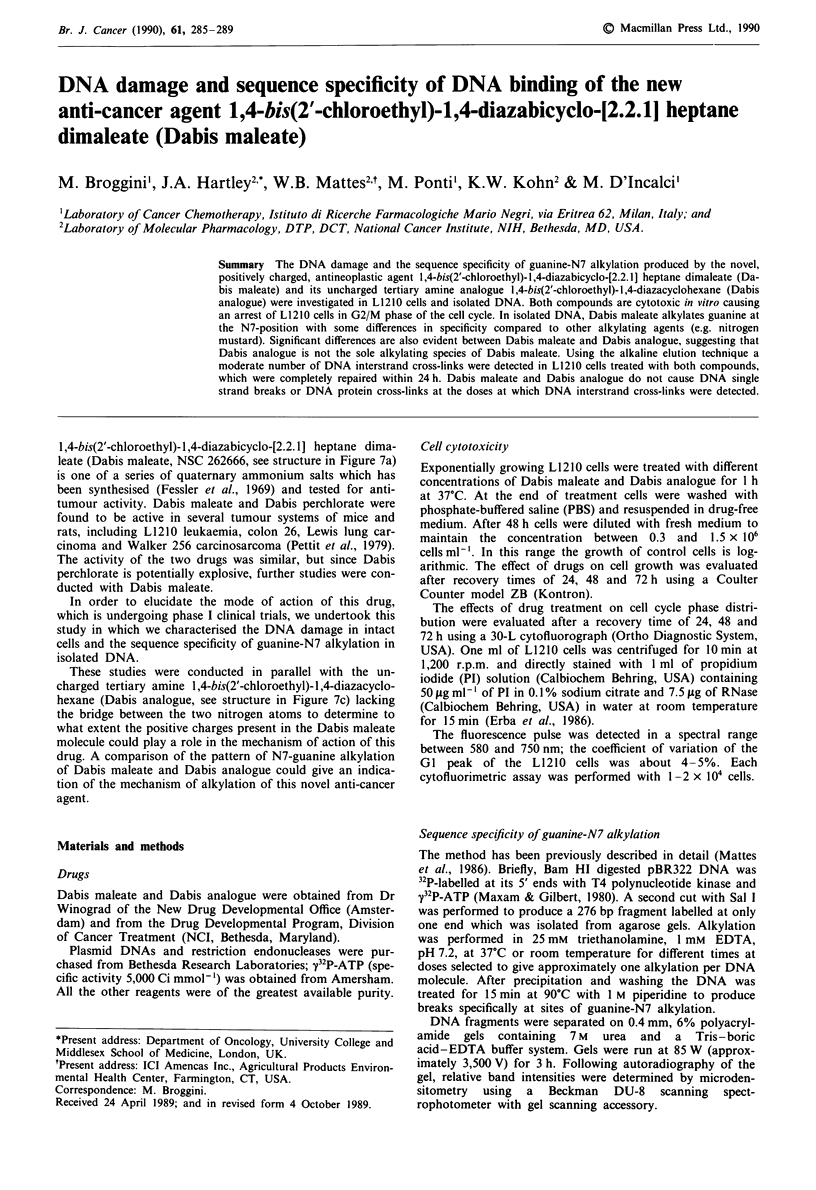

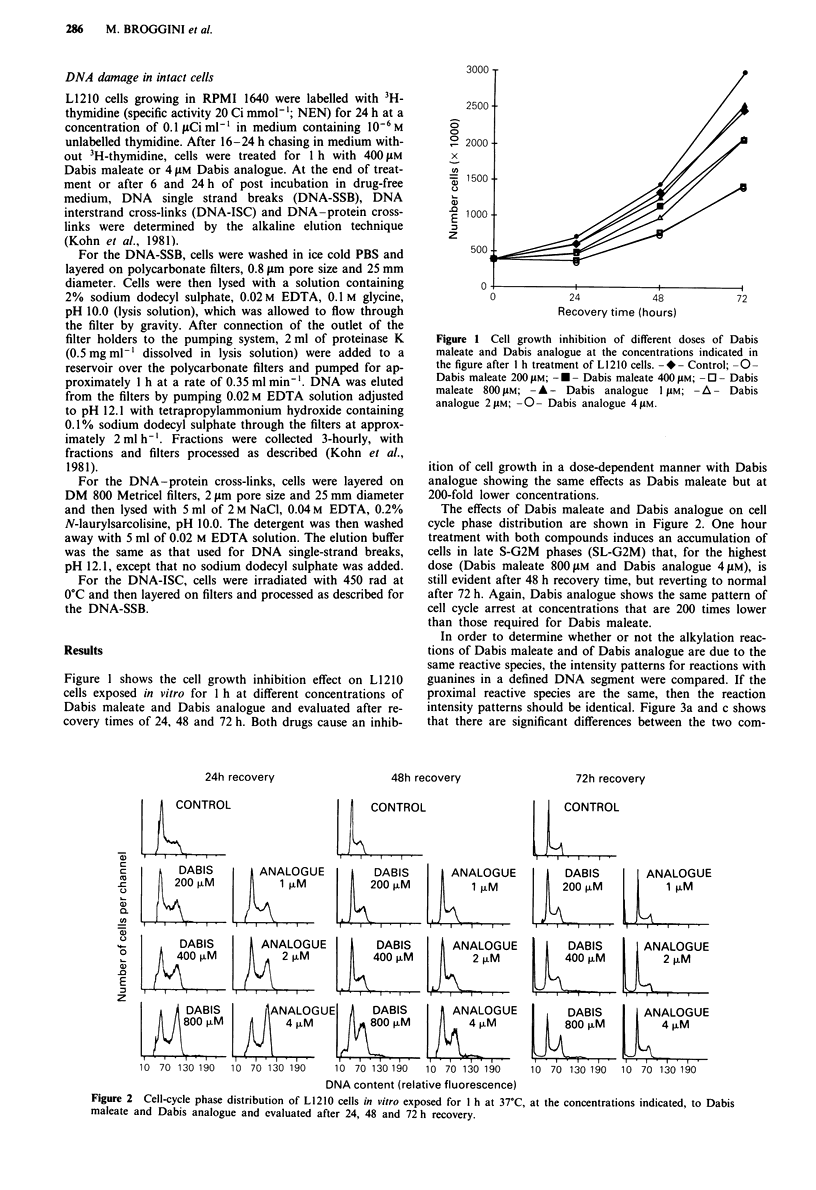

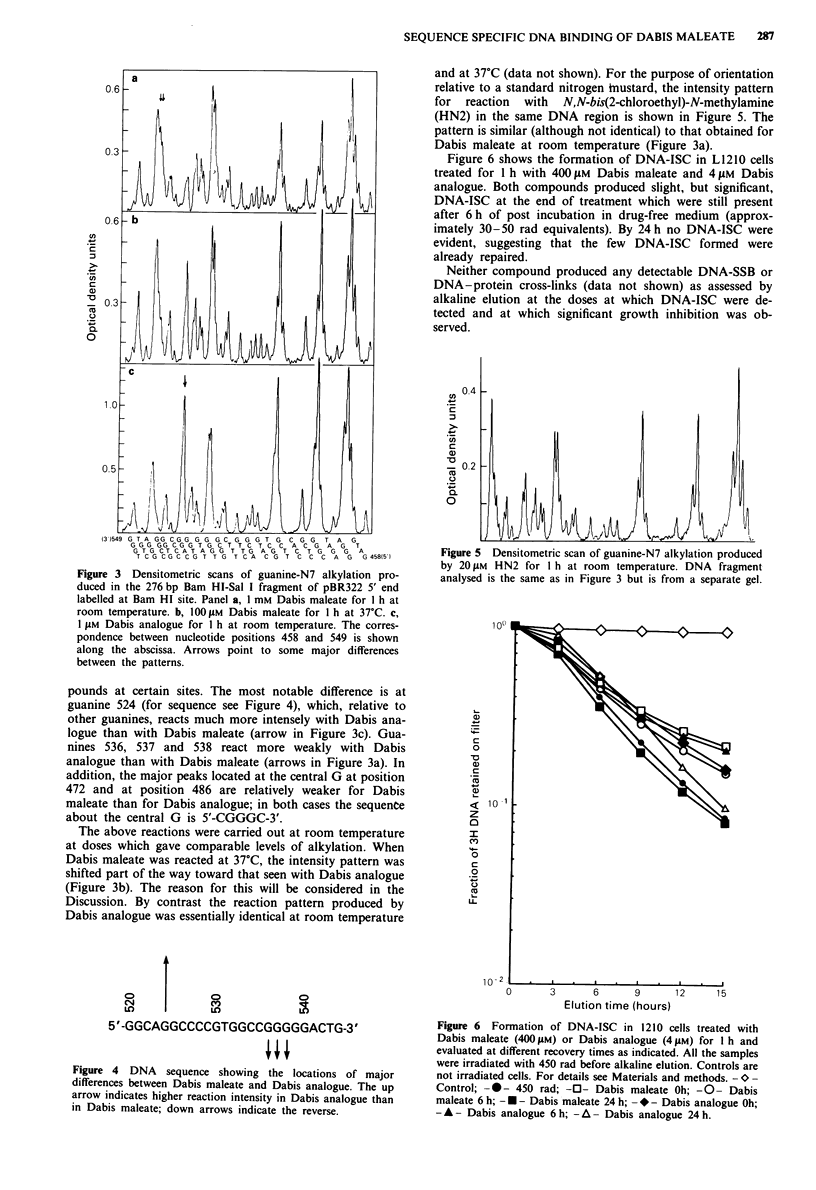

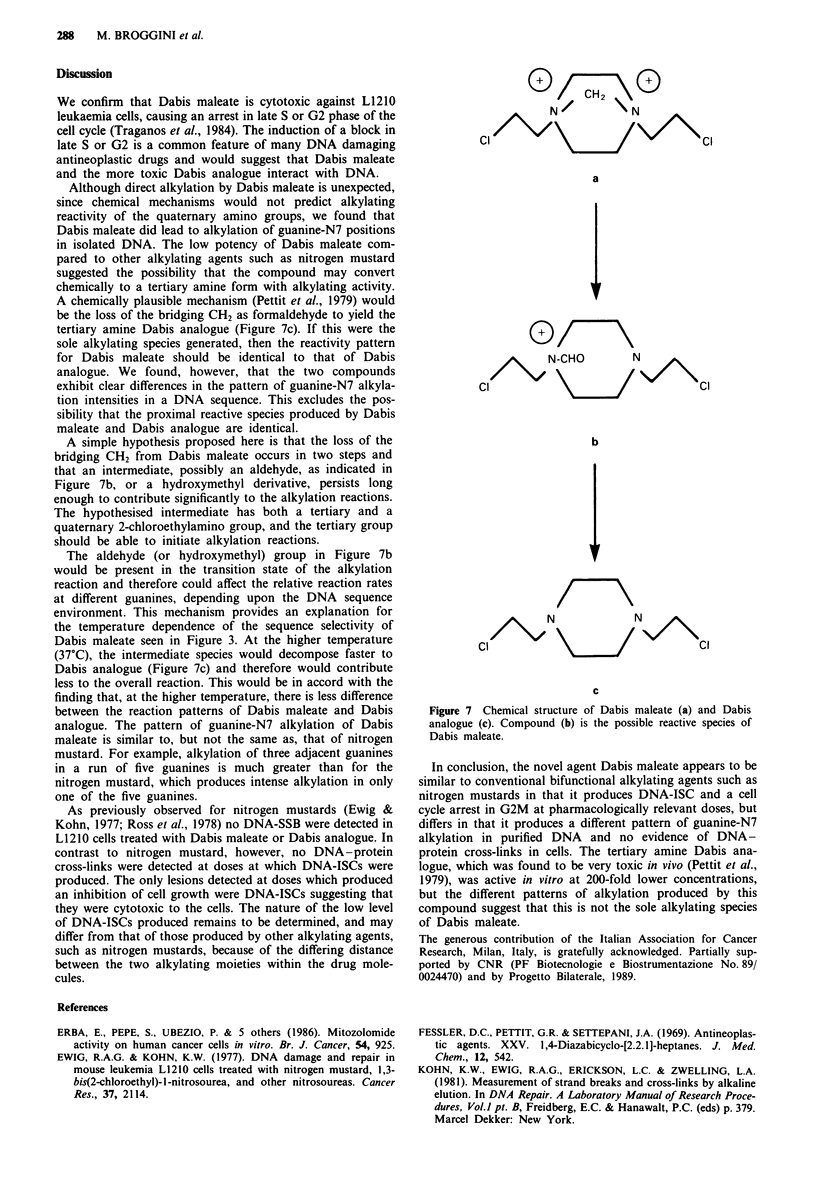

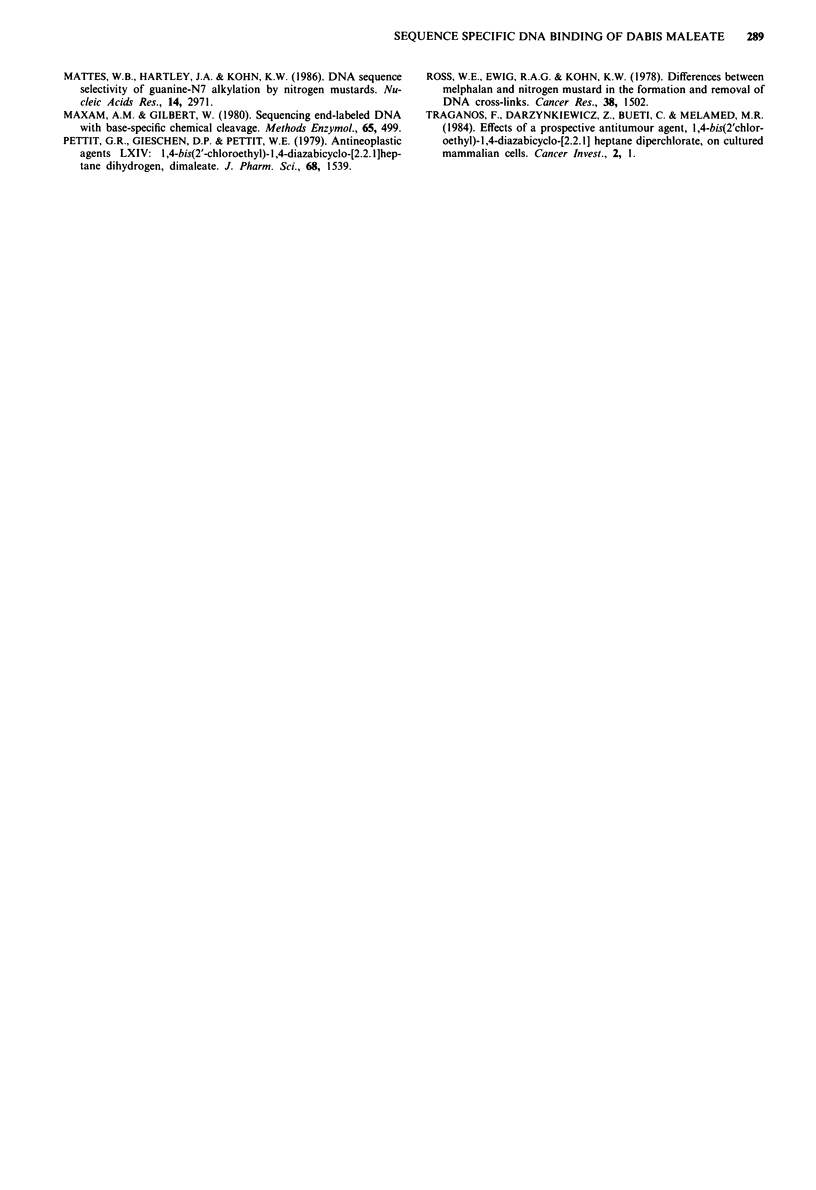


## References

[OCR_00525] Erba E., Pepe S., Ubezio P., Lorico A., Morasca L., Mangioni C., Landoni F., D'Incalci M. (1986). Mitozolomide activity on human cancer cells in vitro.. Br J Cancer.

[OCR_00528] Ewig R. A., Kohn K. W. (1977). DNA damage and repair in mouse leukemia L1210 cells treated with nitrogen mustard, 1,3-bis(2-chloroethyl)-1-nitrosourea, and other nitrosoureas.. Cancer Res.

[OCR_00534] Fessler D. C., Pettit G. R., Settepani J. A. (1969). Antineoplastic agents. XXV. 1,4-Diazabicyclo [2.2.1] heptanes.. J Med Chem.

[OCR_00548] Mattes W. B., Hartley J. A., Kohn K. W. (1986). DNA sequence selectivity of guanine-N7 alkylation by nitrogen mustards.. Nucleic Acids Res.

[OCR_00553] Maxam A. M., Gilbert W. (1980). Sequencing end-labeled DNA with base-specific chemical cleavages.. Methods Enzymol.

[OCR_00556] Pettit G. R., Gieschen D. P., Pettit W. E. (1979). Antineoplastic agents LXIV: 1,4-Bis(2'-chloroethyl)-1,4-diazabicyclo[2.2.1]heptane dihydrogen dimaleate.. J Pharm Sci.

[OCR_00561] Ross W. E., Ewig R. A., Kohn K. W. (1978). Differences between melphalan and nitrogen mustard in the formation and removal of DNA cross-links.. Cancer Res.

[OCR_00566] Traganos F., Darzynkiewicz Z., Bueti C., Melamed M. R. (1984). Effects of a prospective antitumor agent, 1,4-bis(2'-chloroethyl)-1,4-diazabicyclo-[2.2.1] heptane diperchlorate, on cultured mammalian cells.. Cancer Invest.

