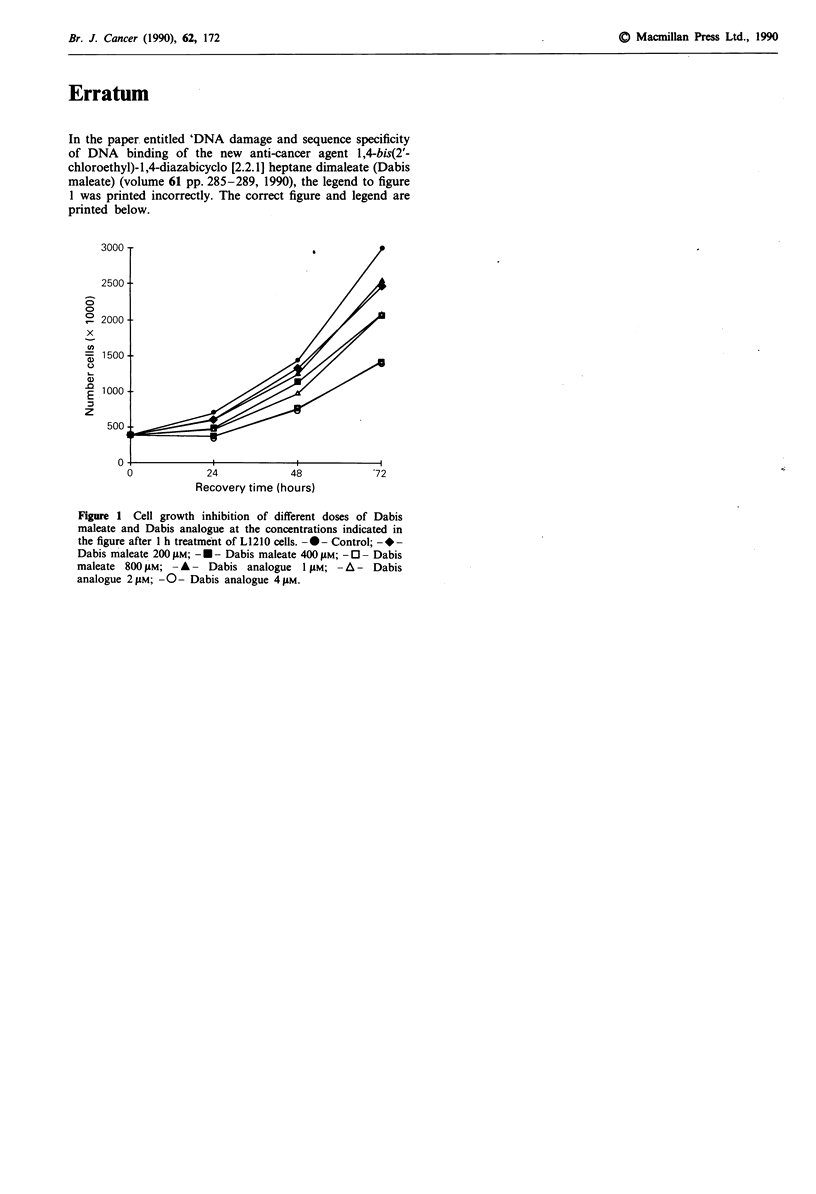# Erratum

**Published:** 1990-07

**Authors:** 


					
Br. J. Cancer (1990), 62, 172                                                                           ?  Macmillan Press Ltd., 1990

Erratum

In the paper entitled 'DNA damage and sequence specificity
of DNA binding of the new anti-cancer agent 1,4-bis(2'-
chloroethyl)-1,4-diazabicyclo [2.2.1] heptane dimaleate (Dabis
maleate) (volume 61 pp. 285-289, 1990), the legend to figure
1 was printed incorrectly. The correct figure and legend are
printed below.

3000
2500

2000
.x

10)

z

o            24            48           '72

Recovery time (hours)

Figure 1 Cell growth inhibition of different doses of Dabis
maleate and Dabis analogue at the concentrations indicated in
the figure after I h treatment of L1210 cells. -0- Control; --
Dabis maleate 200 yMi; -. - Dabis maleate 400 gm; -0 - Dabis
maleate 800 ILM; - A - Dabis analogue 1 gm; - A - Dabis
analogue 2 "Mi; -O - Dabis analogue 4 "M.

Q'I Macmillan Press Ltd., 1990

Br. J. Cancer (I 990), 62, 172